# Team climate and job satisfaction in a mobile emergency service: a multilevel study ^*^

**DOI:** 10.1590/1518-8345.6872.4110

**Published:** 2024-03-15

**Authors:** Hercules de Oliveira Carmo, Marina Peduzzi, Daisy Maria Rizatto Tronchin

**Affiliations:** ^1^ Universidade de São Paulo, Escola de Enfermagem, São Paulo, SP, Brazil.

**Keywords:** Prehospital Care, Patient Care Team, Job Satisfaction, Emergency Medical Services, Ambulances, Nursing, Atención Prehospitalaria, Grupo de Atención al Paciente, Satisfacción en el Trabajo, Servicios Médicos de Urgencia, Ambulancias, Enfermería, Assistência Pré-Hospitalar, Equipe de Assistência ao Paciente, Satisfação no Emprego, Serviços Médicos de Emergência, Ambulâncias, Enfermagem

## Abstract

**Objective:**

to investigate the relationship between team climate and job satisfaction among professionals working in mobile pre-hospital care.

**Method:**

this is a quantitative, correlational study carried out in a mobile pre-hospital care service in the São Paulo Metropolitan Region. The participants were 95 professionals, allocated to 40 teams, who answered three questionnaires: sociodemographic/labor data, Team Climate Scale and S20/23 Job Satisfaction Scale. Descriptive statistics and multilevel linear models were used for the analysis, including moderation effects. The Backward method was used to ascertain the order of significance.

**Results:**

in the models, the relationships between satisfaction with hierarchical relationships and the factor “support for new ideas” moderated for men and “task orientation” for women were significant. For satisfaction with the physical environment, “working hours” and “participation in the team” were significant and, for intrinsic satisfaction, the regime, working hours and the factors “team objectives”, “participation in the team” and “support for new ideas” remained significant, as did the moderation effect between length of service, “participation in the team” and “support for new ideas”.

**Conclusion:**

team climate is influenced by job satisfaction in a heterogeneous way and the moderating effect of this relationship is associated with gender and length of service.

## Resumen

**Objetivo:**

examinar la relación entre el clima en equipo y la satisfacción laboral de los profesionales que trabajan en servicios de atención prehospitalaria móvil.

**Método:**

estudio cuantitativo y correlacional realizado en un servicio de atención prehospitalaria móvil en la Región Metropolitana de São Paulo. Participaron 95 profesionales, distribuidos en 40 equipos, que respondieron a tres cuestionarios: datos sociodemográficos/laborales, Escalas de Clima en Equipo y de Satisfacción Laboral S20/23. Para el análisis, se utilizaron estadísticas descriptivas y modelos lineales multiniveles, incluyendo efectos de moderación. Se empleó el método *Backward* para determinar el orden de significancia.

**Resultados:**

en los modelos, fueron significativas las relaciones entre la satisfacción con las relaciones jerárquicas y el factor “apoyo para nuevas ideas” moderado por el género masculino y “orientación hacia las tareas” en mujeres. Para la satisfacción con el ambiente físico, fueron significativos “jornada laboral” y “participación en el equipo”, y para la satisfacción intrínseca, se mantuvieron significativos el régimen, la jornada y los factores “objetivos del equipo”, “participación en el equipo” y “apoyo para ideas nuevas”, y el efecto de moderación entre el tiempo de actuación, “participación en el equipo” y “apoyo para ideas nuevas”.

**Conclusión:**

el clima en equipo es influenciado por la satisfacción laboral de manera heterogénea y el efecto moderador de esta relación se asocia con el género y el tiempo de actuación en el servicio.

## Resumo

**Objetivo:**

examinar a relação entre clima em equipe e satisfação no trabalho de profissionais atuantes em atendimento pré-hospitalar móvel.

**Método:**

estudo quantitativo, correlacional, realizado em um serviço de atendimento pré-hospitalar móvel da Região Metropolitana de São Paulo. Participaram 95 profissionais, alocados em 40 equipes, os quais responderam a três questionários: dados sociodemográficos/laborais, Escalas de Clima na Equipe e de Satisfação no Trabalho S20/23. Para a análise, foram usados a estatística descritiva e os modelos lineares multiníveis, incluindo efeitos de moderação. Empregou-se o método *Backward* para averiguar a ordem de significância.

**Resultados:**

nos modelos, foram significantes as relações entre satisfação com relações hierárquicas e fator “apoio para novas ideias” moderado ao gênero homem e “orientação para as tarefas” às mulheres. Para satisfação com ambiente físico, foram significantes “jornada de trabalho” e “participação na equipe” e, para satisfação intrínseca, permaneceram significantes o regime, a jornada e os fatores “objetivos da equipe”, “participação na equipe” e “apoio para ideias novas”, e efeito de moderação entre tempo de atuação, “participação na equipe” e “apoio para ideias novas”.

**Conclusão:**

clima em equipe é influenciado pela satisfação no trabalho de modo heterogêneo e o efeito moderador dessa relação associa-se ao gênero e ao tempo de atuação no serviço.

**Highlights:**

**(1)** There was a positive perception of the team climate and job satisfaction.

**(2)**The team climate influenced job satisfaction in a heterogeneous way.

**(3)** The moderating effect of this relationship was associated with gender and working hours.

**(4)** The working regime and working hours directly affected intrinsic satisfaction.

## Introduction

In the context of health care, Mobile Pre-Hospital Care (MPHC) services are designed to provide early assistance, outside the hospital environment, to individuals in urgent and emergency situations at risk of intense suffering, sequelae or death, and to safely refer them to a previously contacted health facility ^(^ ^1^ ^)^. It is recognized that this plays a unique and relevant role in different countries, with improved outcomes, especially in acute diseases and those caused by external causes ^(^ ^2^ ^)^. 

Evidence shows that the presence of integrated and accessible MPHC services and the quality and effectiveness of the care provided by their respective teams have made a significant contribution to reducing avoidable deaths and disabilities ^(^ ^3^ ^-^ ^5^ ^)^. 

In Brazil, in 2003, the Federal Government set up the Mobile Emergency Care Service (MECS), which was designated the central axis for the reorganization of emergency care ^(^ ^1^ ^,^ ^6^ ^)^. Over the course of 20 years (2003-2023), this health facility has expanded in several Brazilian cities. Data shows that between 2015 and 2019, there was an increase in coverage, reaching 85% of citizens in 3,750 municipalities (67.3%) ^(^ ^7^ ^)^. 

The work process at MECS has many peculiarities and is based on two dimensions: assistance and management. The first dimension is made up of professionals from the emergency regulation center (ERC), whose work team is made up of doctors, nurses, medical regulation assistants and radioperators, specialized in answering emergency telephone calls on 192, classifying and prioritizing people’s needs. This dimension also includes the Basic Life Support (BLS) teams, made up of nursing assistants/technicians and first aid drivers, and the Advanced Life Support (ALS) team, made up of doctors, nurses, first aid drivers and pilots ^(^ ^6^ ^)^. These teams are designed to assist individuals with health problems, stabilize them and provide safe, quality referrals to health facilities. 

In terms of management, the managers (service coordinator, technical manager and nursing coordinator) are responsible for carrying out activities aimed at planning, managing the teams, providing logistical support, integrating the service with the other components of the emergency care network (ECN), as well as monitoring quality indicators, promoting training with the emergency education center and evaluating the service from the perspective of workers and users ^(^ ^1^ ^)^. 

Admittedly, this is considered an essential service for the population, endowed with specificities and sensitive to response time, in which effective resolutions of the MPHC depend eminently on effective, collaborative teamwork, permeated by healthy interpersonal relationships and job satisfaction ^(^ ^8^ ^)^. 

Teamwork involves the participation of different professional categories and is characterized by intense interdependence of actions, integration, clarity and recognition of roles, construction of common objectives, sharing of responsibility, joint identity and interprofessional collaboration to provide health results ^(^ ^9^ ^)^. Authors report that among the ways to understand teamwork is to understand and analyze the team climate, defined as “shared perceptions among team members about the policies, practices and procedures of day-to-day patient care” ^(^ ^9^ ^-^ ^11^ ^)^. 

Job satisfaction expresses the worker’s perception of their work, meeting their work values in line with their needs, making it possible to generate feelings of belonging, well-being, pleasure and the establishment of bonds. This can change depending on the intrinsic and extrinsic conditions of both the job and the professional ^(^ ^12^ ^)^. 

The national and international literature shows a close relationship between team climate and job satisfaction, with the former being a relevant predictor of the latter in primary care settings ^(^ ^13^ ^)^ and hospital ^(^ ^14^ ^)^. However, there is a lack of knowledge about teamwork, measured by the constructs “team climate” and “job satisfaction”, as well as their relationship with sociodemographic and work characteristics, from the perspective of workers and teams in the MPHC/MECS. 

Furthermore, it is believed that these constructs contain elements that are linked to work dynamics, interprofessional relationships and the specificities of knowledge among workers, impacting on the quality of care and personal and professional development.

In this way, this research has outlined the following hypotheses: H1 - Team climate influences job satisfaction; H2 - Sociodemographic and work variables are moderators of the relationship between “team climate” and “job satisfaction”.

Considering these premises, the aim of this study was to examine the relationship between team climate and job satisfaction among professionals working in mobile pre-hospital care.

## Method

### Study type

Quantitative study with a correlational design.

### Study setting

This study was carried out at a MECS in a municipality in the Metropolitan Region of São Paulo (SP). Implemented approximately 20 years ago, it had an emergency regulation center (ERC), ten Basic Life Support (BLS) units, three Advanced Life Support (ALS) units, a Rapid Intervention Vehicle (RIV) and four motorcycle ambulances, distributed in 10 decentralized bases in the four regions of the municipality. It should be noted that this service guarantees 100% population coverage and, in 2020, provided a total of 34,070 services (an average of 2,839/month).

### Data collection period

Data collection took place between July and September 2021, following the current regulations regarding health measures due to the COVID-19 pandemic adopted by the National Health Surveillance Agency (Anvisa). It should be pointed out that the data collection was carried out by the researcher and an undergraduate nursing student trained for the activity.

### Population

The population consisted of doctors, nurses, nursing technicians/assistants, medical regulation assistants, radiographers and emergency drivers.

### Selection criteria

The inclusion criteria were professionals who had worked in mobile emergency units and/or ERC for ≥ 6 months and had worked in the same team for ≥ 3 months.

Length of service and working in the same team are relevant factors for understanding and outlining the team climate, especially with regard to creating and establishing bonds and the individual’s commitment to the service and the team; it also encourages sharing experiences, mutual development and fosters interrelationships ^(^ ^10^ ^-^ ^11^ ^)^. 

Workers in administrative positions, coordinators, managers and those on vacation or on leave during the data collection period were excluded.

### Sample definition

The sample was non-probabilistic and of convenience. Of the 185 workers, five were on sick leave, eight were on vacation, 13 had worked for ≤ 6 months and ≤ 3 months in the same team, two worked in the administrative sector and one in coordination. Given the eligibility criteria, 156 workers were eligible and invited to take part in the study, but 34 refused.

Thus, 122 instruments were distributed, but 27 professionals did not return them within the stipulated period. As a result, the sample consisted of 95 professionals (77.8% response rate) belonging to 40 teams (two in the regulation center and 38 in the mobile units).

### Data collection

Three questionnaires were used to collect the data:

#### Sociodemographic and employment data

The variables included were: age, gender, education, type of employment relationship, regime and length of time working at MECS, hours per shift, type of team they were part of, length of time working in the team and other employment relationships.

#### Team Climate Scale (TCS) - Brazilian version

The Brazilian version of the TCS ^(^ ^11^ ^)^ of the original instrument called Team Climate Inventory (TCI) ^(^ ^10^ ^)^, is made up of 38 items divided into four factors: team participation (12 items); support for new ideas (eight items); team goals (11 items); and task orientation (seven items). 

The factors contain affirmative sentences or questions answered using a Likert-type scale, ranging from one to five or one to seven. Thus, in the “participation in the team” and “support for new ideas” factors, there are five response points, ranging from “totally disagree” to “totally agree”. The “team goals” factor contains seven points: 1 and 2 - not at all; 3 to 5 - somewhat; and 6 and 7 - completely. Finally, the “task orientation” factor, also with seven points: 1 and 2 - not much; 3 to 5 - to some extent; and 6 and 7 - a lot.

The overall score for each factor is obtained from the average of the answers, varying according to the number of statements and the degree of ^(^ ^11^ ^)^. 

#### Job Satisfaction Scale S20/23 (EST-S20/23) - Brazilian version

The EST-S20/23, Brazilian version ^(^ ^12^ ^)^ original instrument called *Cuestionario de Satisfacción S20/23* ^(^ ^15^ ^)^, is made up of 20 items, divided into three dimensions: satisfaction with hierarchical relationships (SRH) - 11 items; satisfaction with the physical work environment (SAFT) - five items; and intrinsic job satisfaction (SIT) - four items, answered using a Likert-type scale with five degrees (5 - totally satisfied, to 1 - totally dissatisfied). The average of the sum of the answers gives the overall score for each dimension ^(^ ^12^ ^)^. 

It should be noted that there is no cut-off point for both scales, and the average values that are closest to the estimated maximum for the overall score and for each factor or dimension are considered positive/favorable results ^(^ ^10^ ^-^ ^12^ ^,^ ^15^ ^)^. 

The collection procedure was planned jointly with the service coordinator, discussing the best strategy for approaching and inviting workers to take part in the study.

The professionals were invited individually, in their work environment, and had the option of answering the questionnaires on the spot or handing them in later, with prior arrangements for them to be collected within 15 days. Because of this possibility, the researcher and the undergraduate returned to the field five times, on different days and at different times.

When the questionnaires were being filled in, the collectors kept their distance from the participants, only approaching them to collect the instruments and answer any questions. The completed instruments were stored and protected in sealed envelopes in order to keep the information confidential and later analyzed.

When the questions returned, they were checked to validate that they had been filled in and to avoid unmarked or double-marked questions.

### Data processing and analysis

A database was built using Excel ^®^ software where the variables were entered and, after double-checking, the SPSS ^®^ statistical program, version 20.0, was used for statistical analysis. 

The sample was characterized using descriptive statistics, using summary measures (mean, median, minimum, maximum and standard deviation). The “team climate” and “job satisfaction” were measured by the mean scores of the professionals and teams, and also rescaled to vary on a scale of zero to 100, so that the values obtained represented the same order of magnitude, favoring better interpretation and understanding of the results.

The total internal consistency and the internal consistency between the items of the “team climate” factors and the “job satisfaction” dimensions were assessed via Cronbach’s alpha coefficient in order to measure the reliability and stability of the instruments in the study sample. In this investigation, values > 0.80 were established.

In order to assess the effects of the four team climate factors (independent variable) on each of the three dimensions of job satisfaction (dependent variables), multilevel linear models were adjusted given the hierarchical structure of the allocation of professionals in their respective teams.

Multilevel models consist of a type of regression analysis whose main characteristic is the recognition of the predictive role played by variables from different levels; establishing the assumption that individuals belonging to the same group are subjected to similar stimuli, exerting an influence on them ^(^ ^16^ ^)^. 

In this respect, two levels were considered: level 1 (team climate and satisfaction, analyzed from the professionals’ perspective) and level 2 (team climate and satisfaction, analyzed from the team’s shared perspective).

In the multilevel model, moderation effects were included to assess whether certain sociodemographic characteristics, such as gender (male and female) and age (years), and work characteristics, such as position (emergency regulation center and mobile units), time in the team (between 3 and 12 months, 1 and 3 years, 3 and 5 years and > 5 years) and in the service (between 6 and 12 months, 1 and 3 years, 3 and 5 years and > 5 years), type of team (emergency regulation center, basic life support unit and advanced life support unit), employment relationship (social organization and municipal public), work regime (statutory, consolidation of labor laws and cooperative) and working hours per shift (6 hours, 12 hours, 24 hours and 36 hours), in addition to the four team climate factors, affected the magnitude of the relationship between “team climate” and “job satisfaction”. This model assumes normality in the data, verified using the Kolmogorov-Smirnov test.

The multilevel analysis took place in three stages: in the first, the moderation models for each characteristic were adjusted separately and the effects captured via interaction with the TCS factors. In the second stage, all significant interactions were adjusted; non-significant interactions were excluded using the backward method, and in the third stage multivariate models were adjusted with all the selected characteristics and the significant interactions resulting from the second stage. The significance level adopted was 5%.

### Ethical aspects

The research was approved by the Research Ethics Committee of the School of Nursing of the University of São Paulo, under Opinion nº. 3.890.586, in 2020, and was carried out in accordance with the recommendations of Resolution No. 466/2012 of the National Health Council ^(^ ^17^ ^)^. The workers were invited to take part and were informed about the study’s objectives and how to participate, as well as the risks and benefits of the research. All participants signed the Free and Informed Consent Form. 

## Results

With regard to sociodemographic characteristics, 60 (63.2%) said they were men, 37 (38.9%) had completed higher education. The average age was 44 (SD=9.2) years (min. 23 and max. 61 years) and the median was 45 years. As for education, 40 (64.5%) were predominantly nurses, 22 (35.5%) were nurses, 17 (27.4%) were nursing technicians and 1 (1.6%) was a nursing assistant.

With regard to employment, the majority (61.1%) were municipal civil servants, with an employment contract regulated by the Consolidation of Labor Laws (CLT) (56.8%) and 44.6% had another employment relationship. The average length of time working for MECS was 7.9 (SD=5.6) years (min. 6 months and max. 20 years) with a median of 7.4 years, and the average length of time working for the same team was 3.9 (SD=4) years (min. 4 months and max. 14 years) with a median of 2.4 years.

We found that 89.5% of the professionals worked 12 by 36 hours/shift (first aid drivers, nursing assistants and technicians, nurses and interventional doctors), 3.1% 36 hours/shift (medical regulators), 2.1% 24 hours/shift (interventional doctors) and 5.3% 6 hours a day (medical regulation assistants and radioperators).

Of the total number of professionals, 38 (40%) worked as first aid drivers, followed by 16 nursing technicians (16.8%), 13 nursing assistants (13.7%), 9 nurses (9.5%), 8 interventional doctors (8.4%), 5 medical regulation operator assistants (5.3%), 4 regulatory doctors (4.2%) and 2 radioperators (2.1%).

Regarding the type of teams, it was found that 2 (5%) worked in the ERC, made up of medical regulation assistants, radiographers and medical regulators; 29 (72.5%) in the Basic Life Support unit (BLS), made up of first aid drivers, nursing assistants/technicians; and 9 (22.5%) in the Advanced Life Support unit (ALS), made up of first aid drivers, nurses and medical interventionists.

The reliability of the TCS and EST-S20/23 showed excellent Cronbach’s alpha coefficients - values > 0.90, both overall and between the factors and dimensions.

The teams obtained an average total climate score of 84.9 (SD=9.8) points, ranging from 52.8 to 99.7. With regard to the factors, it was found that “participation in the team” obtained an average of 83.9 (SD=11.1), “support for new ideas” 83.4 (SD=12.1), “team objectives” 85.6 (SD=10.3) and “task orientation” 86.2 (SD=10.8) points.

With regard to the teams’ perception of job satisfaction, the average total score was 89.4 (SD=10.3) points, with a range between 58.3 and 100 points. As for the dimensions, satisfaction with hierarchical relationships (SRH) reached an average of 90.1 (SD =9.2), satisfaction with the physical work environment (SAFT), an average of 87.0 (SD=15.9), and intrinsic job satisfaction (SIT), an average of 90.4 (SD=10.2) points.

The multivariate multilevel models showed that the four “team climate” factors were not simultaneously significant. For the “SAFT” dimension, adjusted by the scores of the factors “support for new ideas”, “team objectives” and “task orientation”, it was noted that the “participation in the team” score was significant (p=0.031). Thus, an increase of 1 point in the score for this factor leads to an increase of 0.045 points in the SAFT score.

Tables 1 , 2 and 3 show the results that remained significant in the multivariate multilevel linear regression models, including the moderation effects of sociodemographic and work characteristics, in order to assess whether these variables affect the magnitude of the relationship between “team climate” and “job satisfaction”. 

**Table 1 -:** Initial and final multivariate multilevel linear regression model for satisfaction with hierarchical relationships among study participants (n=95). São Paulo, SP, Brazil, 2021

	**Initial model**		Final model	
	Adjusted coefficient (95%CI)*	p ^†^	(95%CI)*	p ^†^
**Gender women** (ref. ^‡^=man)	-0,193 (-1,896 to 1,510)	0,824	-0,027 (-1,361 to 1,306)	0,968
**Team climate**				
Participation in the team	0,004 (-0,021 to 0,028)	0,760	-	-
Support for new ideas	0,058 (0,023 to 0,093)	0,001	0,065 (0,030 to 0,100)	<0,001
Team goals	0,017 (-0,006 to 0,040)	0,156	-	-
Task orientation	-0,026 (-0,068 to 0,017)	0,234	-0,004 (-0,045 to 0,038)	0,854
**Climate x gender moderation**				
Support for new ideas	-0,093 (-0,133 to -0,054)	<0,001	-0,083 (-0,130 to -0,036)	0,001
Task orientation	0,081 (0,038 to 0,124)	<0,001	0,070 (0,023 to 0,116)	0,003

^*^ 95%CI = 95% Confidence interval;

^†^ p = p-value at 5% level (p ≤ 0,05)

^‡^ Ref. = Reference

In the final model, the “support for new ideas” factor (p<0.001), the moderation effect between “gender” and “support for new ideas” (p=0.001) and between “gender” and “task orientation” (p=0.003) remained significant.

Thus, it was found that for men, an increase in the “support for new ideas” score leads to an increase in the SRH score (on average 0.065 points more for every 1-point increase in the TCS score for this factor). In women, there was no effect of this factor on SRH (0.065-0.083=-0.018; 95%CI: -0.061 to 0.025; p =0.407).

The TCS factor “task orientation” showed that, in women, an increase in this factor led to an increase in the SRH score (on average 0.070-0.004 = 0.066; 95%CI: 0.039 to 0.093; p<0.001).

**Table 2 -:** Initial and final multivariate multilevel linear regression model for satisfaction with the physical work environment among study participants (n=95). São Paulo, SP, Brazil, 2021

	Initial model Adjusted coefficient (95%CI)*	p ^†^	Final model Adjusted coefficient (95%CI)*	p ^†^
**Shift work** (ref. ^‡^=12 h)		<0,001		<0,001
6 h	-1,049 (-1,536 to -0,563)	<0,001	-1,253 (-1,528 to -0,978)	<0,001
24 h	0,020 (-1,326 to 1,366)	0,977	0,111 (-1,105 to 1,326)	0,858
36 h	0,923 (0,246 to 1,599)	0,007	0,582 (0,222 to 0,942)	0,002
**Team climate**				
Participation in the team	0,041 (-0,005 to 0,087)	0,084	0,076 (0,050 to 0,102)	<0,001
Support for new ideas	0,042 (-0,030 to 0,113)	0,251	-	-
Team goals	0,013 (-0,022 to 0,047)	0,469	-	-
Task orientation	0,002 (-0,046 to 0,051)	0,921	-	-

^*^ 95%CI = 95% Confidence interval;

^†^ p = p-value at 5% level (p ≤ 0.05);

^‡^ Ref. = Reference

The results in Table 2 show that, in the final model, the working hours per shift (p<0.01) and the “participation in the team” factor (p<0.001) remained significant. Thus, professionals working 6-hour shifts had a lower score on the SAFT dimension (on average 1.253 points less) compared to workers on 12-hour shifts. On the other hand, professionals working 36h/shift had higher scores (on average 0.582 points more) than those working 12 h/shift. 

With regard to “participation in the team”, it was found that an increase in this factor leads to an increase in the SAFT dimension score (on average 0.076 points more for every 1-point increase in the TCS score for this factor). No moderation effects were observed between working hours on “team climate” and “job satisfaction”.

Table 3 shows that, in the final model, the work characteristics “work regime” (p=0.015) and “shift work” (p<0.001), the factor “team objectives” (p=0.018), the moderation effects between the factor “participation in the team” and “time at MECS” (p<0.001) and between the factor “support for new ideas” and “time at MECS” (p<0.001) remained significant. As a result, MECS professionals who work under the statutory system have, on average, a lower SIT score of 0.202 points when compared to workers with CLT contracts and cooperative workers. 

**Table 3 -:** Initial and final multivariate multilevel linear regression model for intrinsic job satisfaction among study participants (n=95). São Paulo, SP, Brazil, 2021

	Initial model Adjusted coefficient (95%CI)*	p ^†^	Final model Adjusted coefficient (95%CI)*	p ^†^
**Statutory regime** (ref. ^‡^=CLT ^§^/Cooperators)	-0,239 (-0,457 to -0,022)	0,031	-0,202 (-0,365 to -0,040)	0,015
**Time of MECS** ^||^ (ref. ^‡^=more than 5 years)		0,113		0,419
0,5 |-| 1 ano	3,682 (-0,423 to 7,787)	0,079	3,258 (-0,854 to 7,370)	0,120
1 -| 3 anos	1,438 (0,113 to 2,763)	0,033	0,703 (-0,715 to 2,122)	0,331
3 -| 5 anos	0,207 (-1,765 to 2,179)	0,837	-0,255 (-2,035 to 1,525)	0,779
**Shift work** (ref. ^‡^=12 h)		<0,001		<0,001
6 h	-0,517 (-1,176 to 0,142)	0,124	-0,586 (-1,027 to -0,145)	0,009
24 h	0,355 (0,078 to 0,632)	0,012	0,374 (0,250 to 0,499)	<0,001
36 h	0,633 (0,243 to 1,023)	0,001	0,605 (0,318 to 0,892)	<0,001
**Team climate**				
Participation in the team	0,023 (-0,002 to 0,047)	0,076	0,019 (-0,007 to 0,046)	0,159
Support for new ideas	0,018 (-0,022 to 0,058)	0,376	0,018 (-0,019 to 0,055)	0,341
Team goals	0,023 (0,001 to 0,045)	0,044	0,021 (0,004 to 0,039)	0,018
Task orientation	-0,003 (-0,038 to 0,031)	0,850	-	-
**Moderation team participation x MECS** ^||^ **time**		<0,001		<0,001
0,5 |-| 1 year	-0,186 (-0,288 to -0,084)	<0,001	-0,182 (-0,277 to -0,087)	<0,001
1 -| 3 years	-0,009 (-0,032 to 0,015)	0,473	0,001 (-0,019 to 0,021)	0,917
3 -| 5 years	-0,046 (-0,089 to -0,003)	0,035	-0,024 (-0,060 to 0,013)	0,198
**Moderation support for new ideas x MECS** ^||^ **time**		0,003		<0,001
0,5 |-| 1 year	0,175 (0,075 to 0,275)	<0,001	0,181 (0,086 to 0,275)	<0,001
1 -| 3 years	-0,025 (-0,070 to 0,020)	0,278	-0,020 (-0,066 to 0,025)	0,380
3 -| 5 years	0,061 (-0,011 to 0,133)	0,097	0,041 (-0,017 to 0,098)	0,164

^*^ 95%CI = 95% confidence interval;

^†^ p = p-value at the 5% level (p ≤ 0.05);

^‡^ Ref. = Reference;

^§^ CLT = Consolidation of Labor Laws;

^||^ MECS = Mobile Emergency Care Service

With regard to working hours, professionals working 6 hours/shift had a lower SIT score (on average 0.586 points) when compared to those working 12 hours. On the other hand, workers who worked 24 and 36-hour shifts had higher scores (on average 0.374 and 0.605 points more, respectively) than those who worked 12 hours/shift.

With regard to “team objective”, it was observed that an increase in the score for this factor leads to an increase in the SIT score (on average 0.021 points more for every 1-point increase in the factor mentioned).

When moderating the “participation in the team” factor, it was found that for professionals who had worked with the MECS for up to 12 months, an increase in the score on this factor led to a reduction in the SIT dimension score, on average -0.182+0.0190 = -0.163 (95%CI: - 0.259 to - 0.067; p=0.001) points less for every 1-point increase in the score on this factor.

As for the “support for new ideas” factor, it was found that for professionals who had worked at MECS for up to 12 months, an increase in this factor led to an increase in the SIT score, on average 0.181+0.018 = 0.199 (95%CI: 0.114 to 0.283; p<0.001) points more for every 1-point increase in the team climate score.

## Discussion

The findings of this research confirm that “team climate” influences “job satisfaction”, but in a heterogeneous way. Similar results were found in studies of primary health care workers in Brazil ^(^ ^13^ ^)^, obtaining a significant and favorable relationship between the two constructs. In addition, an increase in the score for the “team goals” factor led to an increase in the score for “intrinsic job satisfaction” and “physical environment”; and “team goals” and “task orientation” to an increase in the score for “satisfaction with hierarchical relationships”. 

At the international level, a study conducted in intensive care units of a public hospital in Greece, with the participation of nurses, showed that the factors “support for new ideas”, “team goals” and “task orientation” were influenced by aspects of job satisfaction, such as the relationship with the supervisor/manager, the nature of the work and communication ^(^ ^14^ ^)^. 

Thus, it can be corroborated that teamwork has potential and can improve job satisfaction among professionals/workers and, consequently, provide better results in health care for users, families and the community ^(^ ^9^ ^)^. 

With regard to the analysis of the association between “team climate” and “job satisfaction”, moderated by sociodemographic and work characteristics, it was found that for men, an increase in the score for the factor “support for new ideas” led to greater satisfaction, in relation to hierarchical relationships; for men, the category of first-aid drivers predominated. On the other hand, for women, the largest contingent of the nursing team, an increase in the “task orientation” factor raised the SRH score.

With regard to innovation, or “support for new ideas”, authors describe it as an important measure of team performance, since it relates to the ability to respond to demands or “pressures” at work, proposing a new way of carrying out activities, in a pro-social environment, characterized by mutual help and information sharing ^(^ ^18^ ^)^. 

In this context, it is possible to infer that the workers pointed out that the teams were looking for new answers, collaborating with each other to explore new ideas and dedicating the necessary time to put them into practice. In addition, they had the support of their superiors and the possibility of making autonomous decisions at work, participating in decisions at the institution, and they considered the way in which agreements were made to be appropriate in the light of labor legislation.

A multicenter study to explore the climate in health and social care teams in thirteen European countries identified high scores for the factor “support for new ideas”. The main related factors were sufficient time to work in a constructive and solution-oriented way, management support and freedom to innovate ^(^ ^19^ ^)^. 

A study with nurses from a MECS in a city in the state of Paraná described the need to mobilize inventive intelligence, i.e. improvisation, in the face of divergences between the prescribed and actual work imposed on MPHC professionals, in order to get closer to the necessary demands of the task. It also revealed that an effective interpersonal relationship with the team was a facilitating factor in the work process ^(^ ^20^ ^)^. 

A study carried out with interprofessional teams in Chile, aimed at examining satisfaction with the team, climate and leadership, showed the contributions of the younger generation, the use of creativity and innovation in the search for better health outcomes. In addition, it indicated that the presence of transformational leaders in teams strengthens well-being, favors dialogue, manages tensions and supports innovation ^(^ ^21^ ^)^. 

As for the “task orientation” factor, this encompasses characteristics related to working with teammates, marked by the exchange of ideas and practical support, monitoring among professionals to produce quality care, the practice of reflection and criticism on the work carried out in favor of better results and the existence of clear criteria that the team seeks to meet in order to achieve excellence in health care ^(^ ^9^ ^,^ ^19^ ^,^ ^22^ ^)^. To do this, they rely on the members of the SRH. 

The development of teamwork requires a solid commitment on the part of all members and requires a set of attributes, such as: shared responsibility, interprofessional communication, fairness in decision-making, guidance and supervision, psychological safety and periodic moments for sharing ^(^ ^23^ ^)^. 

Spaces for debate and collective deliberation are a common practice among MPHC workers, as they find in this resource the trust, security and support they need to practice their knowledge, jointly drawing up rules and guidelines for the development of activities, in order to achieve better results ^(^ ^24^ ^)^. 

A study carried out in a MECS in the state of Goiás, Brazil, with nursing technicians, 61.2% of whom were female, found that the nature of the work, contentment with the activities performed, associated with positive relationships with supervisors, were the domains that showed the greatest job satisfaction ^(^ ^25^ ^)^. 

A systematic review aimed at examining the predictors of greater job satisfaction and engagement among APH professionals revealed that leadership behavior, support and exchange with experienced colleagues were positively associated with job satisfaction ^(^ ^26^ ^)^. 

Specifically in relation to the association between “team climate” and the SAFT dimension, the working hours per shift remained significant (lower score for 6-hour shifts compared to 12-hour shifts; and higher score for 36-hour shifts compared to 12-hour shifts) and the “participation in the team” factor.

It is recognized that the physical work environment at MECS is diverse and takes place in what can be called micro-environments, such as the ERC, the decentralized base, the mobile emergency unit and the scene of the incident. Furthermore, job satisfaction in these contexts involves aspects of physical structure, ventilation, air conditioning, hygiene and cleanliness.

In this study, the professionals working 6-hour shifts correspond to the category of medical regulation assistants and radioperators, and 36-hour shifts (medical regulators), both of whom work in the ERC; and those working 12/36 hours are the workers in mobile emergency units (emergency drivers, nursing assistants and technicians, nurses and interventional doctors), which allows us to infer that the relationship between working hours and SAFT stems, among other factors, from the type of team and the professional category (doctors) who are allowed to work 24 and 36 hour shifts with rest breaks.

The literature shows that the work environment in the ERC imposes challenges and, consequently, can cause dissatisfaction. A systematic review study on this site pointed to threats to physical health related to shift work, outdated and ergonomically poorly adjusted equipment, isolation, high noise levels and inadequate lighting. Mental health risks included exposure to traumatic liaisons or verbally aggressive people, practically non-stop work in high-pressure environments, an excessive number of shifts and a lack of support from leadership ^(^ ^27^ ^)^. 

When evaluating the working environment of MECS professionals in Fortaleza, Ceará, the findings showed that of the 10 items covering the physical working environment, nine were classified as critical and one as serious, posing a risk to people’s safety ^(^ ^28^ ^)^. 

With regard to the “participation in the team” factor, which led to an increase in the SAFT score, it appears that this relates to the sharing of information, understanding and acceptance of members, interactions, collaboration and listening, as well as formal and informal meetings.

In the MPHC, communication permeates the entire dynamics of the work process, including the ERC, care and management, and the relationship with patients/users. This is an essential tool, capable of fostering trust between team members, to ensure the quality of care. It is therefore vital that the information shared is objective and clear, so that teams can recognize situations and prepare to provide effective care ^(^ ^29^ ^)^. 

In this respect, it is worth highlighting the results of a study with MPHC professionals in the southeastern United States, which showed that communication between members of the ERC and ambulance teams was fundamental for developing a shared understanding of the patient’s condition, for carrying out direct assessment and immediate care in cases of stroke ^(^ ^30^ ^)^. 

With regard to the relationship between “team climate” and SIT, it was found that MECS professionals working under the statutory system had a lower score in this dimension when compared to workers with contracts regulated by the CLT and cooperative workers.

In this study, statutory workers accounted for 40% of the workforce, and it can be inferred that, because they have greater stability in the institution and are used to the activities carried out, they analyze the institutional context more realistically, with repercussions on dissatisfaction with the intrinsic aspects of the job. On the other hand, those with CLT contracts, most of them on a temporary basis, given the scenario presented at the time (pandemic), may have felt challenged and motivated to carry out their activities, envisioning professional opportunities to develop skills and competencies and gain respect and trust from their team and managers.

Research involving MPHC technicians and paramedics showed that the lack of opportunities, career progression, benefits and promotions, plus dissatisfaction with professional fulfillment, were the motivating and driving factors behind the intention to leave the service ^(^ ^31^ ^)^. 

The “team objective” factor was significant in the SIT dimension, since an increase in the climate score implied an increase in satisfaction. Thus, the more the team’s objectives are clear, appropriate and shared among the members, the greater the professional’s satisfaction with their own work, with the opportunities it offers to collaborate and excel, with meeting and achieving objectives and targets.

MPHC professionals work in dangerous environments and under unpredictable conditions. This work requires critical, quick and assertive thinking, while providing life-saving care ^(^ ^8^ ^,^ ^20^ ^)^. Clarity of objectives and the purpose of the work among team members are predictors of an effective team ^(^ ^9^ ^)^. 

In Sweden, a study showed that the inability of coworkers and the lack of knowledge and common goals within the team were reasons for dysfunctional cooperation between nursing teams in an MPHC service. Furthermore, these points led professionals to a fragmented understanding of the patient’s condition, the objectives and the care to be provided, generating feelings of frustration and loneliness, as well as an overload of activities in the team ^(^ ^32^ ^)^. 

Also in SIT, there was a moderation in the climate factor “participation in the team” among professionals who had worked at MECS for up to 12 months. The increase in the score implied a reduction in the score for the “job satisfaction” dimension. There was moderation in the climate factor “support for new ideas” and time at MECS, since an increase in this factor in professionals with up to 12 months at MECS led to an increase in the SIT score.

Given this result, it is conjectured that professionals with less time at MECS are willing to learn, gain more experience, seek interaction, contribute and participate in the team, using their skills and abilities, giving up, at this point, issues related to work, in terms of being an element that fosters fulfillment.

It is therefore understood that for workers with less time at MECS, the more support there is for creativity, innovation and encouragement to transform the work environment, the greater the job satisfaction with the intrinsic factors - fulfillment, meaning, opportunity, visibility and cooperation.

Research with MPHC professionals from the German emergency medical system found that workers with up to five years of ambulance work, aged between 18 and 40 and recent graduates had better job satisfaction scores ^(^ ^33^ ^)^. 

A study aimed at elucidating the professional experiences of ambulance nurses found, from the reports, that the coexistence of nurses with less time in the ambulance with more experienced and competent ones had a positive effect on the work of the younger ones, marked by elements such as competence, skill, meaning of work and feeling of security in professional practice ^(^ ^34^ ^)^. 

In view of the above, the innovative nature of this research stands out, as it investigates the relationship between “team climate” and “job satisfaction” in a MECS, using multilevel linear models, since there is a lack of studies adopting such analyses.

The limitations lie in the size of the sample and the type of team, made up mostly of two members, making it impossible to analyze by grouping to select teams with contrasting profiles. Another limitation is the lack of research using the TCS and EST - S20/23 in the context of the MECS, restricting discussion of the findings and signaling the need for future studies in this area of health care.

However, the research makes significant contributions to support the management of MPHC services, since it presents the intervening elements that permeate the relationship between “teamwork climate” and “job satisfaction” and which can enhance or limit the practice environment, impact the organization and interpersonal relationships, as well as personal and professional development and the qualification of care.

## Conclusion

The hypotheses of this investigation were confirmed, showing that team climate influences job satisfaction, and the moderating effect of this relationship is associated with gender, in terms of satisfaction with hierarchical relationships, and with length of service for intrinsic satisfaction.

In addition, the working day directly affects satisfaction with the physical environment, and the working regime and working day have an impact on intrinsic satisfaction.

These elements constitute opportunities for improvement, with the aim of fostering a climate that is favorable to teamwork, providing job satisfaction and qualifying care in emergency pre-hospital assistance.
